# Uninvited Guest: Arrival and Dissemination of Omicron Lineage SARS-CoV-2 in St. Petersburg, Russia

**DOI:** 10.3390/microorganisms10081676

**Published:** 2022-08-20

**Authors:** Anna Gladkikh, Vladimir Dedkov, Alena Sharova, Ekaterina Klyuchnikova, Valeriya Sbarzaglia, Tatiana Arbuzova, Majid Forghani, Edward Ramsay, Anna Dolgova, Anna Shabalina, Nadezhda Tsyganova, Areg Totolian

**Affiliations:** 1 Saint Petersburg Pasteur Institute, Saint Petersburg 197101, Russia; 2 Martsinovsky Institute of Medical Parasitology, Tropical and Vector Borne Diseases, Sechenov First Moscow State Medical University, Moscow 119435, Russia; 3 Krasovskii Institute of Mathematics and Mechanics, Yekaterinburg 620108, Russia

**Keywords:** SARS-CoV-2, COVID-19, Omicron BA.1, Omicron BA.2, Russia

## Abstract

Following its emergence at the end of 2021, the Omicron SARS-CoV-2 variant rapidly spread around the world and became a dominant variant of concern (VOC). The appearance of the new strain provoked a new pandemic wave with record incidence rates. Here, we analyze the dissemination dynamics of Omicron strains in Saint Petersburg, Russia’s second largest city. The first case of Omicron lineage BA.1 was registered in St. Petersburg on 10 December 2021. Rapid expansion of the variant and increased incidence followed. The peak incidence was reached in February 2022, followed by an observed decline coinciding with the beginning of spread of the BA.2 variant. SARS-CoV-2 lineage change dynamics were shown in three categories: airport arrivals; clinical outpatients; and clinical inpatients. It is shown that the distribution of lineage BA.1 occurred as a result of multiple imports. Variability within the BA.1 and BA.2 lineages in St. Petersburg was also revealed. On the basis of phylogenetic analysis, an attempt was made to trace the origin of the first imported strain, and an assessment was made of the quarantine measures used to prevent the spread of this kind of infection.

## 1. Introduction

The COVID-19 pandemic, ongoing for the last 2.5 years, is caused by the SARS-CoV-2 virus, a member of the *Coronaviridae* family, genus *Betacoronavirus*. COVID-19 is highly contagious, and clinical manifestations of infection vary from asymptomatic to severe forms of pneumonia or death. As of 22 June 2022, the SARS-CoV-2 virus has caused 538,321,874 confirmed infections and 6,320,599 deaths worldwide [1]. For the entire pandemic period in Russia to date, there have been 18,406,485 cases, including 380,643 fatalities [2].

SARS-CoV-2 appears to be an actively evolving virus. Some variants, due to their high transmissibility and epidemic potential, are classified by the World Health Organization (WHO) as variants of concern (VOC) and require increased vigilance by health authorities. At certain stages of the pandemic, strains of various lineages were given VOC status: Alpha (B.1.1.7); Beta (B.1.351); Gamma (P.1); Delta (B.1.617.2); and Omicron (B.1.1.529).

First isolated on 11 November 2021 in Botswana (Southern Africa), the Omicron strain caused an explosive increase in incidence and rapidly spread globally, displacing the previously dominant Delta variant [3], despite the fact that 70% of the population in the developing/developed world were fully vaccinated [4]. Currently, Omicron is the dominant globally circulating variant, accounting for >98% of viral sequences shared on GISAID after February 2022 [5]. The Omicron variant has been shown to be able to evade immunity induced by those vaccines used until recently [6].

The Omicron variant (B.1.1.529) is currently an exceptional lineage in terms of the number of mutations. It has more than 50 different mutations, the majority of which are related to the spike glycoprotein (about 30 SNPs, 3 deletions, 1 insertion). Cumulatively, these may underlie reduced antibody/SARS-CoV-2 binding seen among convalescents or those vaccinated [7]. It has been shown that the Omicron spike protein (S) is less efficiently cleaved by furin and less fusogenic than that of the Delta variant [8,9].

Since its appearance, the Omicron lineage has undergone evolution, giving rise to several discrete sublines with a characteristic set of mutations. According to the WHO, the Omicron VOC lineage currently includes the BA.1, BA.2, BA.3, BA.4, and BA.5 lineages, as well as BA.1/BA.2 recombinants [5]. BA1 and BA2 are the most frequently detected variants. As the first Omicron variant to appear, BA.1 rapidly spread around the world and became the dominant variant. However, BA.2 replaced BA.1 as the dominant epidemic subvariant in more and more countries over time [10].

In the context of the generally tense epidemiological situation globally, enhanced continuous monitoring of viral incidence and circulating genetic variants is carried out in Russia in accordance with World Health Organization (WHO) recommendations. The first Omicron cases were registered on 6 December 2021 in two Russians arriving in Moscow from South Africa [11]. In Russia’s Northwestern Federal District, against the backdrop of high incidence by the end of 2021, Omicron prevalence reached 2.1% [12].

Saint Petersburg, Russia’s second largest metropolis featuring a high population density, is home to Pulkovo International Airport. Without the strictest of quarantine measures, Pulkovo will most likely become a gateway for the importation of new genetic variants into the city and their further distribution. Our study is devoted to describing the distribution dynamics and variability of the Omicron BA.1 and BA.2 variants in St. Petersburg, as well as to trace the origin of the first imported strain, while assessing the effectiveness of the applied quarantine measures in preventing the spread of this kind of infection.

## 2. Materials and Methods

### 2.1. Study Materials

Nasopharyngeal swabs of patients with diagnosed COVID-19 during the period 29 November 2021 to 1 May 2022 were collected and delivered to the Saint Petersburg Pasteur Institute for sequencing and further genetic study. These were collected at St. Petersburg hospitals, clinics, and at Pulkovo Airport (St. Petersburg) from arrivals. From hospitals and clinics, 10% of all positive samples were sent to the Institute for investigation. All COVID-19 positive samples from arrivals at Pulkovo Airport with clinical manifestations, as well as without (voluntarily agreeing to examination), were sent to the Institute. Swabs were collected in 500 µL of special transport medium or phosphate-buffered saline (pH 7.0) and stored at −20 °C until further analysis. A total of 25,470 samples were examined, of which: 684 samples were obtained from persons arriving at Pulkovo Airport; 16,425 samples were from outpatients; and 8361 samples were from hospitalized patients in St. Petersburg.

### 2.2. RNA Purification

Total nucleic acid samples were obtained by extraction and purification using the QIAamp Viral RNA Extraction Kit (QIAGEN, Hilden, Germany) with the QIAcube Connect automatic station (QIAGEN, Hilden, Germany). Samples were eluted with 50 µL of AVE Buffer (QIAGEN, Hilden, Germany) and stored at −70 °C until molecular analysis.

### 2.3. RT-qPCR Assay for Detection of SARS-CoV-2 BA.1 and BA.2 Lineages

For screening of Omicron variants in the St. Petersburg population, an RT-PCR assay was developed. Initially, primers and probes were developed to detect the BA.1 lineage using a specific region (deletion 211 and insertion 214 in the S protein gene). By 7 February 2022, primers and probes were also developed to detect the BA.2 lineage (using deletion 24–26 in the S gene). Oligonucleotide sequences are presented in Table 1. The total volume of reaction mix for samples was 25 µL containing the following: 1 µL of BioMaster Mix (Biolabmix, Novosibirsk, Russia); 12.5 µL of 2× reaction buffer (Biolabmix, Novosibirsk, Russia); 0.25 µL of each primer and probe (final concentration of 0.4 µM for primers and 0.28 µM for probes); and 10 µL of RNA sample. The amplification program was as follows: 50 °C for 15 min; 95 °C for 5 min; followed by 40 cycles (95 °C for 10 s and 55 °C for 30 s). Fluorescence was registered during the 55 °C step in HEX (for BA.1) and FAM (for BA.2). Reactions were performed using the CFX96 thermocycler (BioRad, Hercules, CA, USA). The fluorescence threshold was established as the middle value in the linear increase range of the positive control’s fluorescence graph (in log units). Amplification results were considered positive if the level of fluorescence crossed the threshold. As an RT-PCR control, an external positive control for PCR (C^+^) was applied [13]. In addition, negative controls for extraction (Ex^−^) and PCR (C^−^) were used to exclude false positives due to potential cross-contamination.

### 2.4. Primer Design and Reverse Transcription

In order to obtain near-complete SARS-CoV-2 genomic sequences (excluding 5′ and 3′ ends), a total of 138 primer pairs were designed using the Primal Scheme web-based design tool [14]. The pairs produce 300–320 nt products with 50 nt overlaps (Supplementary Table S1). All SARS-CoV-2 sequence variants present in GISAID (as of 28 May 2021) were considered at the moment of primer design. Reverse transcription used random hexamers and the Reverta-L Kit (AmpliSens^®^, Moscow, Russia) following the manufacturer’s protocol. Samples (cDNA) were stored at –70 °C until amplification.

### 2.5. Multiplex PCR

Genome-spanning primers from the designed primer panel were diluted to a concentration of 10 pmol/mL and combined into six pools. Every pool included every 6th primer in the list; each pool included 23 primer pairs. Hot-start multiplex amplification reactions were performed in a 25 μL total volume containing 2 μL of cDNA, 0.1 μM of each primer, and 12.5 μL of 2× BioMaster HS-Taq PCR mix (Biolabmix, Novosibirsk, Russia). The following thermal cycling parameters were used: 95 °C for 3 min; 35 cycles (93 °C for 10 s, 57 °C for 30 s, 72 °C for 30 s); and a final extension (72 °C for 5 min). Reactions were performed in a C1000 Touch thermocycler (Bio-Rad, Hercules, CA, USA). Products were analyzed by 2.0% agarose gel electrophoresis in the presence of ethidium bromide. Amplified fragments were mixed, then cleaned by the AMPure XP Purification Kit (Beckman Coulter, UK) according to the manufacturer’s instructions (1:1 sample:beads ratio). Concentrations of the fragment mixes were measured with a Qubit 4.0 fluorimeter (Invitrogen, Waltham, MA, USA) using the dsDNA HS Assay Kit (Invitrogen, Waltham, MA, USA) and then used for library preparation.

### 2.6. Library Preparation and Sequencing

Amplicons were diluted to 200 ng/µL and used as input for each library preparation reaction. Library preparation was performed according to the Illumina TruSeq Nano DNA Kit protocol with the TruSeq DNA CD Indexes Kit (Illumina Inc., San Diego, CA, USA). Briefly, amplicons were subjected to a series of enzymatic reactions: end repair; adenylation; and ligation of adapter sequences at the 5′ and 3′ ends. Products were then amplified by 8 cycles of PCR according to the protocol. The resulting libraries were purified using Illumina Sample Purification Beads and eluted in 50 µL of resuspension buffer. Quality assessment of final libraries was carried out on the QIAxcel Advanced capillary system (QIAGEN, Hilden, Germany); fragment sizes (amplicon insert plus sequencing adapters) were about 420–450 bp. All libraries were quantified using the Qubit 4.0 fluorimeter and the Qubit dsDNA HS Assay Kit (Invitrogen) prior to sequencing. Each library was diluted to 10 nM. The same volume from each 10 nM library was taken to obtain an equimolar pool of 96 libraries. The resulting pool was denatured and diluted to a final library concentration of 8 pM. Sequencing was performed on the MiSeq instrument using MiSeq V3 chemistry.

### 2.7. Genome Assembly

The quality of Illumina reads was assessed using the FastQC program [15]. Raw reads were filtered with Trimmomatic [16] to remove adapters, low-quality nucleotides, and biased sequences at the ends of reads (parameters ILLUMINACLIP:TruSeq3-PE.fa:2:30:10:2 SLIDINGWINDOW:4:20 HEADCROP:30 MINLEN:50). Genome assembly was carried out by mapping to the SARS-CoV-2 reference genome (Wuhan-Hu-1 strain, NCBI accession number NC_045512.2) using Bowtie 2 [17]. For variant calling and consensus generation, samtools and bcftools software were used [18]. The Nextclade tool was used to assess the quality of assembled sequences and to assign genomes to lineages [19]. Sequences were uploaded to GISAID under the following IDs: EPI_ISL_12949381-EPI_ISL_12949587; EPI_ISL_12953196-EPI_ISL_12953203; EPI_ISL_12875298-EPI_ISL_12875386; and EPI_ISL_13032194-EPI_ISL_13032293.

### 2.8. Phylogenetic Tree Reconstruction

For phylogenetic reconstruction, all sequences (744) belonging to the Omicron BA.1 lineage with sample collection dates from 24 November 2021 to 7 December 2021 were downloaded from the GISAID database. To reduce the data set, duplicate genomes and sequences with high homology (≥99%) were clustered with CD-hit [20]; 134 genomes were selected for tree construction. Genomes were aligned with MAFFT v7.453 [21]. Ends (5′, 3′) were trimmed, and the phylogenetic tree was constructed with IQ-TREE [22]. The workflow was tree reconstruction with ultrafast bootstrap (1000 replicates); the JC substitution model was used. The dendrogram was visualized with iTOL v.6 [23]. A global phylogenetic tree of SARS-CoV-2 variants was constructed using the tools implemented in Nextclade [19].

### 2.9. Network Reconstruction

Phylogenetic network analyses were performed with Network v.10.2.0.0 using the median-joining algorithm [24]. The samples used for input were 215 BA.1-strain S gene sequences and one of the first Omicron strains from South Africa (EPI_ISL_6913991, collected 9 November 2021). The epsilon parameter was set to 0 or 10, resulting in two similar networks. Network calculation can generate unnecessary median vectors and links. To clean up the network, post-processing MP calculation was performed. The network further was annotated and customized using node pie chart coloring to indicate groups of collected samples. The tree output was chosen for display of results.

## 3. Results

### 3.1. Analysis of Omicron Lineage Dynamics in St. Petersburg

From 29 November 2021 to 9 December 2021, the BA.1 variant was not detected in St. Petersburg. Partial S gene sequencing confirmed that isolated strains belonged to the Delta lineage (presence of L452R and E478K substitutions along with the EF156-157 deletion). The first BA.1 case in St. Petersburg was registered in a woman arriving at Pulkovo Airport from the United Arab Emirates on 10 December 2021. Between 13 and 20 December, Omicron infections were observed in passengers arriving from Amsterdam and Frankfurt. Up to the end of 2021, cases of Omicron strain importation to St. Petersburg from various countries of Europe and Africa, as well as Turkey, were registered. In this time, the share of Omicron infections exceeded 50% of all COVID-19 detections in arrivals.

**Figure 1:**
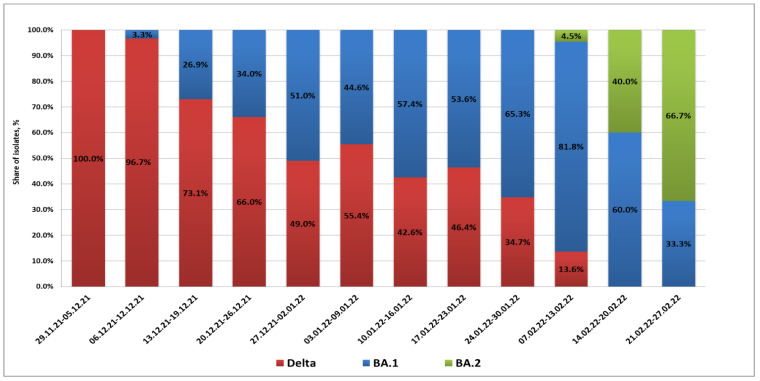
**Viral lineage dynamics in Pulkovo Airport arrivals with a confirmed COVID-19 diagnosis.** Weekly dynamics of SARS-CoV-2 sublineages in arrivals (29 November 2021 to 27 February 2022) are shown.

In 2022, there was a further increase in the share of imported cases associated with Omicron strains. The maximum percentage of BA.1 strains (81.8%) was registered in the second week of February. At the same time, the appearance of BA.2 strains in arrivals was already being noted; their share among positive samples was 4.5%. A concurrent, rapid decrease in imported Delta strains was seen. In mid-February, the profile of SARS-CoV-2 strains imported through the airport included only strains from Omicron lineages BA.1 and BA.2. The proportion of BA.2 strains continued to grow, and they exceeded 60% by the end of February (Figure 1).

A weekly analysis of COVID-19 morbidity in St. Petersburg showed a rapid increase starting from 145.9 per 100,000 population in early January and reaching 2409.5 in February. Starting from the second half of February, incidence began to decline sharply. Analysis of outpatient samples received in January 2022 showed a rapid increase in the share from the Omicron BA.1 lineage and a decrease in the share from Delta. Hence, from the first week of 2022, the share of Omicron was 13.6%, but by the fourth week, this figure reached 84.5%. It should be noted that mild clinical severity was more often observed with Omicron infections compared with those caused by Delta.

**Figure 2:**
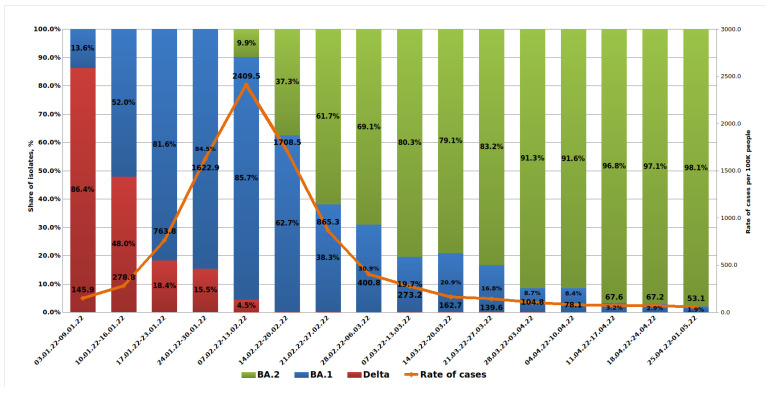
**Viral lineage dynamics in outpatients with a confirmed COVID-19 diagnosis.** Weekly dynamics of SARS-CoV-2 sublineages in outpatients, and COVID-19 morbidity in St. Petersburg (3 January 2022 to 1 May 2022), are shown.

The first BA.2 lineage strain was detected in an outpatient (sample H261) on 15 January 2022. Later in January, sporadic detections of the BA.2 lineage were seen in outpatients using partial S gene sequencing to detect its characteristic mutation profile. By the time the test system was introduced into routine monitoring in the second week of February, the share of BA.2 variants in outpatients had reached 9.9%. Further increases in BA.2’s contribution were observed with almost complete displacement of the Delta lineage. Since mid-February, the Delta variant has been encountered only sporadically in outpatients. The share of the BA.1 variant continued to decline. By the end of March, it was already less than 10% (Figure 2).

In hospitalized patients with a diagnosis of moderate or severe COVID-19, the increase in the share of BA.1 occurred more smoothly. Thus, by the end of January, BA.1 strains represented 57.2%. The first BA.2 detection in a hospitalized patient in St. Petersburg was registered on 21 January 2022 (sample H402, Pokrovskaya Hospital, St. Petesburg, Russia). By the time the test system was introduced into routine monitoring, the share of BA.2 strains had reached 10%, while BA.1 dominated, and the share of Delta strains accounted for less than 1%.

**Figure 3:**
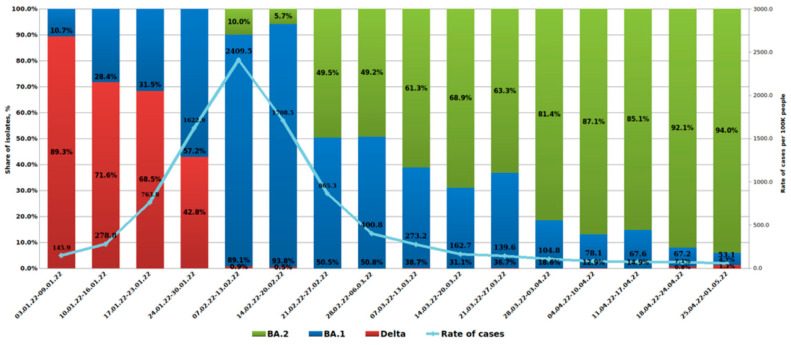
**Viral lineage dynamics in hospitalized patients with a confirmed COVID-19 diagnosis.** Weekly dynamics of SARS-CoV-2 sublineages in inpatients, and COVID-19 morbidity in St. Petersburg (3 January 2022 to 1 May 2022), are shown.

By the end of February, there was a sharp increase in the share of BA.2 strains among inpatients. A subsequent, further increase in the share of BA.2 was observed, which was smoother compared to the trend in outpatients. By the beginning of May, the share of BA.2 was 94%, and BA.1 accounted for only 4.7%. It should be noted that the contribution of Delta strains to the structure of inpatient morbidity was higher, amounting to 1.3% by the beginning of May (Figure 3).

### 3.2. Analysis of Omicron BA.1 Genetic Diversity in St. Petersburg

For the period from December 2021 to January 2022, 215 SARS-CoV-2 whole genome sequences of strains belonging to the BA.1 lineage were obtained. Of these, 52 were from those who arrived at Pulkovo Airport from abroad, 142 were from patients at outpatient clinics, and 21 were from hospital patients. Those in the latter two groups (outpatients, inpatients) indicated no recent travel history or contact with those arriving from abroad in their clinical interview (anamnesis). According to the Pangolin nomenclature, the strains were assigned to sublineages: BA.1 (60 strains); BA.1.1 (102 strains); BA.1.1.1 (2 strains); BA.1.1.11 (1 strain); BA.1.1.14 (1 strain); BA.1.1.15 (3 strains); BA.1.14 (6 strains); BA.1.15 (14 strains); BA.1.15.1 (1 strain); BA.1.17 (5 strains); BA.1.17.2 (16 strains); BA.1.18 (1 strain); and BA.1.20 (3 strains). The number of SNPs compared to the reference strain ranged from 50 to 61.

The first Omicron strain in St. Petersburg, brought in by a passenger arriving from the United Arab Emirates (UAE, 10 December 2021), belonged to the BA.1.1 sublineage, which is characterized by the presence of an R346K mutation in the S gene. The BA.1.1 sublineage also includes isolates identified during a dormitory outbreak at St. Petersburg Polytechnic University. A total of 20 sequences were received from dormitory students or their contacts.

In addition to R346K, some other sublineages within the Omicron clade are also characterized by specific amino acid substitutions. Sublineages BA.1.17 and BA.1.17.2 are characterized by the presence of a V1887I substitution in the ORF1a reading frame. The BA.1.17.2 sublineage, which included 16 strains, is characterized by an A701V mutation (S gene). Sublineages BA.1.15 and BA.1.15.1, which included 15 strains, are characterized by D343G (N gene) and L106F (ORF3a).

In addition to 30 known S gene SNPs [25], solitary mutations were identified in positions E132Q, S162I, M177I, P230Q, I231L, T284S, N354H, A672V, S686R, I1081V, D1163Y, and D1260G. Additional substitutions were also found to be present in two or more strains. The A67V substitution characteristic of Omicron strains was found only in 118 strains. Other findings were: two strains lacked T95I (S gene); one strain carried T95V; nine strains lacked D339G; one strain lacked E484A; and one strain carried E484V. An uncharacteristic substitution for Omicron, F643L, was found in 16 strains; the characteristic H655Y substitution was seen only in 169. The absence of a mutation in the N679K position was noted in 18 strains; P681H was absent in 20 strains. The A701V mutation was detected in 31 strains. In addition to those belonging to BA.1.17.2, it was noted in a number of strains in other BA.1 sublineages (Table 2).

**Figure 4:**
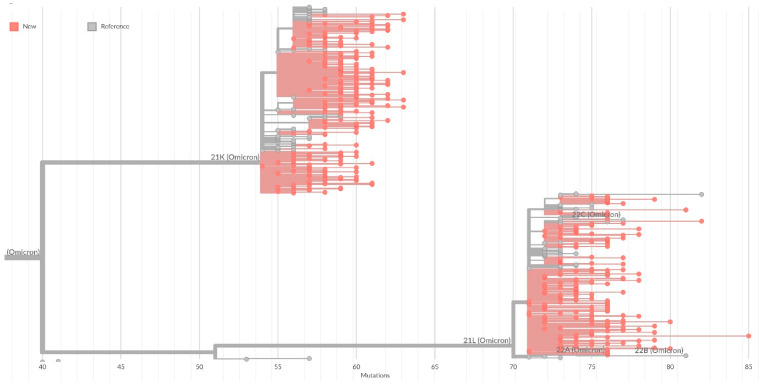
**SARS-CoV-2 phylogenetic tree reconstruction of Omicron branches based on Nextclade tools.** Strains obtained within this work are in red; strains from Nextstrain database are gray. The scale is in substitutions per site compared to the Wuhan-Hu-1/2019 reference sequence (GenBank: MN908947). 21K in Nextstrain lineage corresponds to BA.1 Pangolin lineage, 22 L lineage corresponds BA.2, 22A lineage corresponds BA.4, 22 B lineage corresponds BA.5, 22C lineage corresponds BA.2.12.1.

On the general tree of Nextstrain strains, all identified strains were included in the 20K cluster (Omicron), with the formation of subclusters corresponding to sublineages within BA.1 (Figure 4).

### 3.3. Clonality and Diversity of the SARS-CoV-2 Omicron BA.1 Lineage in St. Petersburg

The genomic variation of Omicron BA.1 lineage from St. Petersburg was analyzed based on S gene alignments with the median-joining network algorithm developed to reconstruct the shortest phylogenetic tree from a given data set. The analysis identified 20 clusters, and the rest formed distinct nodes. Nodes differed from each other by one or two nucleotide substitutions. Five main clusters (100, 165, 175, 145, SARS-CoV-2) include strains brought to St. Petersburg through Pulkovo Airport arrivals. They form clonal complexes with strains identified among patients in the city. The first strain, A26, imported from the UAE, along with strains from Turkey and Serbia, is included in the biggest cluster (cluster 100). The cluster also included strains isolated during the COVID-19 outbreak caused by Omicron at the St. Petersburg Polytechnic University dormitory (December 2021) and strains from inpatients. Clusters 165 and 175 also included strains from all three sample categories. Cluster 145 mainly included strains from those coming from the UK or the Netherlands.

**Figure 5:**
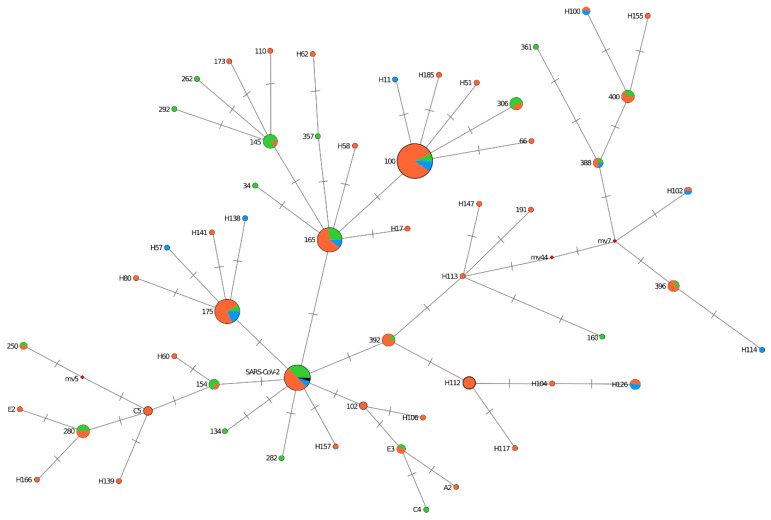
**Phylogenetic network of SARS-CoV-2 Omicron BA.1 S gene sequences.** Circle areas are proportional to the number of taxa, whereas each tick on the links represents a mutated nucleotide position. The median-joining network algorithm (epsilon parameter set to 10) was employed. Clusters are named by one of the sequences forming it. The node pie chart coloring illustrates the proportion of each group in the node. Key: strains introduced to St. Petersburg by Pulkovo Airport arrivals—green; strains from outpatients—orange; strains from inpatients—blue; and South African reference strain (EPI_ISL_6913991)—black.

In addition to large clonal complexes, smaller ones were formed on the tree, and some strains also formed individual nodes. Used as a reference, one of the first Omicron strains from South Africa (EPI_ISL_6913991) was included in the large mixed SARS-CoV-2 cluster, including strains brought from the UK, the Dominican Republic, and the Republic of Belarus (Figure 5).

To clarify the origin of the A26 strain, a phylogenetic reconstruction was carried out with strains from GISAID isolated within three weeks before the first imported case in St. Petersburg. On the dendrogram, the A26 strain (brought from the UAE) clusters together with two strains from Austria and is included in one cluster with a strain from Israel. The phylogenetic tree contains clusters that combine strains from individual countries (for example, South Africa, England), as well as numerous clusters that combine strains isolated in several remote regions. Examples of geographically widespread clusters include: one that combines strains from South Africa, USA, and the Netherlands; and one grouping strains from South Africa, several European countries, and Australia (Supplementary Figure S1).

### 3.4. Analysis of Omicron BA.2 Genetic Diversity in St. Petersburg

In January–March (2022), 189 BA.2 lineage sequences were obtained in St. Petersburg. The first BA.2 strain was detected in patient H261, whose sample was taken for laboratory analysis on 15 January 2022. The strains were attributed to sublineages as follows: BA.2—157 strains; BA.2.10—6 strains; BA.2.12—1 strain; BA.2.23—11 strains; BA.2.3—1 strain; BA.2.9—9 strains; and XQ—1 strain (a BA.1.1/BA.2 recombinant lineage with an S gene mutation profile characteristic of BA.2 strains). The number of SNPs, compared to the reference strain, ranged from 57 to 80.

In addition to 28 characteristic S gene mutations, solitary mutations were identified: L5F; del69-70; H69Y; V90I; A262G; R567K; N679K; V772I; P812L; S943R; S943R; A944S; P1143S; and P1162S. A number of strains lacked S gene substitutions typical for BA.2 strains at: position 27 (11 strains); position 440 (2 strains); position 655 (2 strains); and position 681 (2 strains). Additional substitutions were also found in sublineage BA.2.9 at positions F490S (five strains) and I1227V (two strains) (Table 3). On the general tree of Nextstrain strains, all identified strains were included in cluster 20L (Omicron) (Figure 2).

## 4. Discussion

Based on currently available data, the overall risk associated with Omicron, despite an observed decline in COVID-19 incidence, remains very high. To date, this is the only circulating variant of concern monitored by the WHO [5]. A number of studies have confirmed that the Omicron variant has a significantly greater ability to evade the immune response. Vaccinated individuals and convalescents are easily reinfected, which is unlike other SARS-CoV-2 strains [26,27,28,29]. Omicron also has a significant advantage in transmissivity. After emergence in South Africa, within several weeks it spread exponentially worldwide; Omicron’s speed was faster than other previous variants [30].

The first Omicron strain was detected in St. Petersburg (lineage BA.1) on 10 December 2021, a month after the first case was detected globally, despite the introduction of a quarantine in Russia for passengers arriving from African countries. Further rapid spread of Omicron strains followed in the city’s population. The previous variant, Delta, had dominated over the previous 6 months, and its spread was impressive. The first Delta cases were registered in April (2021), and such strains totally displaced the others by July (2021) [12]. Omicron’s dominance required less time than Delta’s. Indeed, the Omicron variant supplanted Delta in less than two months. By February (2022), its share in the viral structure already exceeded 95% in the city.

The emergence and spread of the Omicron variant gave rise to a new wave of COVID-19 featuring a record-high level of incidence compared to the previous level. It reached more than 24,000 new cases per day in St. Petersburg, despite the restrictive measures taken to control the spread of the new variant. Thus, in 2021, the maximum morbidity in Russia’s Northwestern Federal District was recorded in November, amounting to 1185.8 per 100,000 throughout the region [12]; morbidity in February in St. Petersburg specifically exceeded 5000 cases per 100,000. At the same time, the proportion of mild or asymptomatic cases was nearly 61% at the end of 2021 [27], yet reached 78% by February 2022. The proportion of severe cases decreased and did not exceed 1% of total cases. Several reports have ascribed lesser Omicron severity to lower lung involvement in comparison with the Delta variant, alongside higher replication in bronchi [31]. Analysis of incidence and transmission data confirms that Omicron is more contagious than Delta, while most cases are mild or asymptomatic.

The COVID-19 incidence peak in St. Petersburg coincided with the appearance of the BA.2 variant. Next, in the context of rapid displacement of BA.1, there has been a sharp decline in incidence. It is likely that the BA.2 lineage is less contagious, or more likely to cause an asymptomatic course. This reduces the detection rate, yet the risk of transmission remains high due to a potential lack of symptoms or interest in seeking medical intervention.

An analysis of BA.1 diversity showed the presence of several clonal complexes. These include both imported strains and strains from those without a history of international travel. This suggests that the spread of the Omicron lineage in St. Petersburg occurred due to multiple imports. Several factors likely indicate that it is impossible to stop the spread of this kind of respiratory viral infection in the conditions of a modern metropolis in the absence of strict quarantine measures. These factors are high transmissivity, high viral variability, and the existence of multiple contacts.

After confirming the discovery of a new viral variant, designated Omicron, Russia and a number of countries globally suspended air traffic or introduced quarantine measures for citizens arriving from African countries. However, analysis of the phylogenetic relationships among the first detected strains (November to early December 2021) showed the presence of mixed clusters. Monitoring of Pulkovo Airport arrivals by country revealed that, in the absence of direct flights from African countries, arrivals with Omicron strains were mainly from Europe and Asia (Supplementary Table S3). The first variant was reported from a passenger arriving from the UAE, but the strain clusters with isolates from Austria. In conditions of high human mobility, viral variants spread rapidly. Given Omicron’s higher transmissivity compared to previous SARS-CoV-2 variants, infection can occur at any stage of the route. Thus, regional entry restrictions, and increased control of arrivals from only selected countries, are ineffective measures to prevent the importation and spread of new SARS-CoV-2 genetic variants.

Global Omicron transmission began in November–December 2021 with the BA.1 lineage, soon to be replaced by BA.2 featuring spike protein differences [32]. A recent study shows that the later-appearing BA.2 lineage spread faster than BA.1 [33]. Starting from mid-January 2022, BA.2 cases were registered in St. Petersburg; they later practically replaced BA.1. According to a WHO report (15 May 2022), the Omicron VOC is the dominant variant circulating globally, accounting for nearly all sequences reported to GISAID. The dominant lineage is BA.2, and its descendant sublineages (i.e., BA.2.X) account for up to 97% of sequences submitted to GISAID as of May 2022. Other genetic variants have further declined in global prevalence, falling below 1% [34]. These include BA.1 and its sublineages (BA.1.X, etc.), BA.3, and Delta variants.

Omicron’s competitive advantages were initially provided by a large number of mutations in the S protein and its receptor-binding domain (RBD). In addition, the Omicron variant mutated and evolved during its early transmission. During its first 47 days of spread, 398 nucleotide substitutions and 51 deletions/insertions were identified in Omicron sequences [30]. Mutation R346K spread rapidly, leading to the first sublineage, BA.1.1 [35]. Initially, R346K (characteristic of Mu VOC strains) was considered an additional BA.1 mutation, occurring in 33.9% of strains (49,609 BA.1 sequences) [36]. Among the strains obtained in this work, sublineage BA.1.1 with its characteristic R346K mutation is widespread, accounting for nearly 50%. R346K is located in the RBD and has been shown to affect binding between SARS-CoV-2 and class II antibodies, somewhat weakening neutralization effects [37,38].

The seven Omicron mutations previously characteristic of the other four VOCs suggest a possible recombinational origin of Omicron [36]. The L5F mutation, characteristic of Iota variants, and relatively rare in Omicron strains, was found in two St. Petersburg strains. Mutations that appeared in the first weeks of Omicron circulation are the A701V mutation (characteristic of the Beta variant) and the F643L mutation, located near the S1/S2 cleavage site. They may be associated with enhanced fusogenicity and pathogenicity of SARS-CoV-2 variants [39]. In this work, F643L was found in 16 strains, and A701V was found in 31 strains. Among St. Petersburg strains, L452R was not found; its presence in Omicron strains may provide evasion from cellular immunity and increased infectivity [40,41]. On the other hand, the contribution of some mutations to weakening of the immune response can be overestimated due to the presence of antigenemia as a bias factor in immunodiagnostic assays [42].

BA.2 strains showed fewer additional mutations. One of them, F490S (in two strains), was previously identified in the RBD of Lambda strains, apparently improving immune evasion [41]. Mutation S704L (located in spike protein S2 region) is rare, and its exact impact on the virus has not been well studied. L452Q and S704L are key sites defining the signature of sublineage BA.2.12.1 [43], which was not identified within the obtained sequences.

Given the high variability seen among strains, and the recent emergence of new Omicron sublineages, it is clear that the evolution of this lineage is ongoing. Each successive subvariant has seemingly become better at human transmission and antibody evasion [44,45]. Monitoring of genetic variants through sequencing and genomic analysis of SARS-CoV-2 strains has been, and remains, the recommended measure to combat COVID-19. Despite the presence of dominant variants of concern, the emergence of new local variants is possible at any time. As such, it is necessary to be vigilant for their timely detection.

## 5. Conclusions

The sharp rise in COVID-19 incidence observed in St. Petersburg at the beginning of 2022 was due to the rapid expansion of Omicron lineage BA.1. Moreover, its uninvited arrival in the city has a multiple-import signature. A decline in COVID-19 incidence coincided with the emergence and spread of lineage BA.2. Regional restrictions in the fight against highly contagious SARS-CoV-2 strains showed low efficiency, and they were unable to prevent the rapid spread of Omicron in St. Petersburg. Continued monitoring of SARS-CoV-2 variant succession, including genomic analysis to identify or track mutations, remains critically important. Such data are needed to further our understanding of viral evolution and to develop appropriate measures to prevent pandemic burdens and complications.

## Figures and Tables

**Table 1:** Primers and probes for detection of Omicron variants.

Name	Oligonucleotide Sequence (5′–3′)	Target
OBA1-F	ATA TAT TCT AAG CAC ACG CCT AT	Del211 Ins214EPE
OBA1-R	ACC TAG TGA TGT TAA TAC CTA TTG	
OBA1-prb	ATAGTGCGTG AGCCAGAAGA TCTCgc	
OBA2-F	TCA GTG TGT TAA TCT TAT AAC CAG	del24-26
OBA2-R	AGA ACA AGT CCT GAG TTG AAT G	
OBA2-prb	ACC AGA ACT CAA TCA TAC ACT AAT TCT TTC	

**Table 2:** Mutations registered in two or more BA.1 genomes from St. Petersburg.

S Gene Amino Acid Substitution	Number of Strains
L5F	2
A67V A67A	97 118
T95I T95T	213 2
I145D I145I	213 2
G339D G339G	206 9
R346K	108
N354H N354S	1 1
E484E E484V	1 1
F643L	16
H655Y H655H	169 46
N679K N679N	197 18
P681H P681P	195 20
A701V	31

Note: only nonsynonymous substitutions were considered for analysis.

**Table 3:** Mutations registered in two or more BA.2 genomes from St. Petersburg.

S Gene Amino Acid Substitution	Number of Strains
A27S A27A	178 11
N440K N440N	187 2
F490S	5
H655Y Y655Y	182 7
P681H P681P	187 2
S704L	2
I1227V	2

Note: only nonsynonymous substitutions were considered for analysis.

## Supplementary Materials

The following supporting information can be downloaded at: https://www.mdpi.com/article/10.3390/microorganisms10081676/s1, Figure S1: Phylogenetic tree of Omicron variants available in GISAID from 24.11.21 to 07.12.21. The phylogenetic tree was constructed using 135 complete genome sequences, excluding 5′ and 3′ ends. Numbers at the nodes show bootstrap values. The JC nucleotide substitution model was used; Table S1: Primer panel designed for the study; Table S2: SARS-CoV-2 variants used for phylogenetic tree; Table S3: Passenger information for confirmed Omicron BA.1 cases.
