# Supplementary material for: Uninvited Guest: Arrival and Dissemination of Omicron Lineage SARS-CoV-2 in St. Petersburg, Russia
Source: Microorganisms. 2022 Aug 20;10(8):1676. doi: 10.3390/microorganisms10081676 (PMC9414241; doi:10.3390/microorganisms10081676)
Supplement: Supplementary file 1 [file microorganisms-10-01676-s001.zip › Table S3.pdf]

**Table S3. Passenger information for confirmed Omicron BA.1 cases.**

| Sample number | Date of delivery to the laboratory | Date of arrival (dd.mm.yyyy) | Arrival from |
|---|---|---|---|
| 133 | 23.12.2021 | n/d | Great Britain |
| 134 | 23.12.2021 | n/d | Africa |
| 145 | 27.12.2021 | n/d | Great Britain |
| 158 | 27.12.2021 | 25.12.2021 | Spain |
| 160 | 27.12.2021 | 24.12.2021 | Germany |
| 165 | 27.12.2021 | 25.12.2021 | Spain |
| 170 | 27.12.2021 | n/d | Great Britain |
| 179 | 28.12.2021 | 26.12.2021 | France |
| 181 | 28.12.2021 | 26.12.2021 | Great Britain |
| 34 | 16.12.2021 | 15.12.2021 | flight from Amsterdam |
| 35 | 16.12.2021 | 15.12.2021 | flight from Frankfurt |
| 72 | 20.12.2021 | n/d | Netherlands |
| 73 | 20.12.2021 | n/d | England |
| 76 | 20.12.2021 | 18.12.2021 | Great Britain |
| 77 | 20.12.2021 | 18.12.2021 | United Arab Emirates (UAE) |
| A26 | 10.12.2021 | n/d | arrival from the UAE<br>**first Omicron case in St. Petersburg** |
| C4 | 30.12.2021 | 28.12.2021 | Turkey |
| G2 | 30.12.2021 | n/d | n/d |
| 244 | 06.01.22 | 04.01.2022 | Dominican Republic |
| 247 | 06.01.22 | n/d | n/d |
| 257 | 06.01.22 | 05.01.2022 | Serbia |
| 262 | 08.01.22 | 05.01.2022 | UAE |
| 278 | 08.01.22 | 06.01.2022 | Turkey |
| 280 | 08.01.22 | n/d | n/d |
| 282 | 08.01.22 | 06.01.2022 | Turkey |
| 288 | 08.01.22 | 07.01.2022 | Hungary |
| 289 | 08.01.22 | 07.01.2022 | Israel |
| 292 | 08.01.22 | 06.01.2022 | Turkey |
| 302 | 09.01.22 | 07.01.2022 | Dominican Republic |
| 305 | 09.01.22 | 07.01.2022 | Georgia |
| 306 | 09.01.22 | 07.01.2022 | Dominican Republic |
| 316 | 10.01.22 | 08.01.2022 | Poland |
| 342 | 11.01.22 | 09.01.2022 | Cuba |
| 348 | 11.01.22 | 09.01.2022 | Egypt |
| 350 | 11.01.22 | 09.01.2022 | Russia |
| 355 | 11.01.22 | 09.01.2022 | Egypt |
| 356 | 11.01.22 | 09.01.2022 | Russia |

| | | | |
|---|---|---|---|
| 357 | 11.01.22 | 09.01.2022 | Turkey |
| 367 | 11.01.22 | 10.01.2022 | Russia |
| 370 | 11.01.22 | 10.01.2022 | Russia |
| 377 | 11.01.22 | 10.01.2022 | Belarus |
| H236 | 16.01.22 | 17.01.2022 | Egypt |
| H237 | 16.01.22 | n/d | n/d |
