# Supplementary figures and images for: Uninvited Guest: Arrival and Dissemination of Omicron Lineage SARS-CoV-2 in St. Petersburg, Russia
Source: Microorganisms. 2022 Aug 20;10(8):1676. doi: 10.3390/microorganisms10081676 (PMC9414241; doi:10.3390/microorganisms10081676)

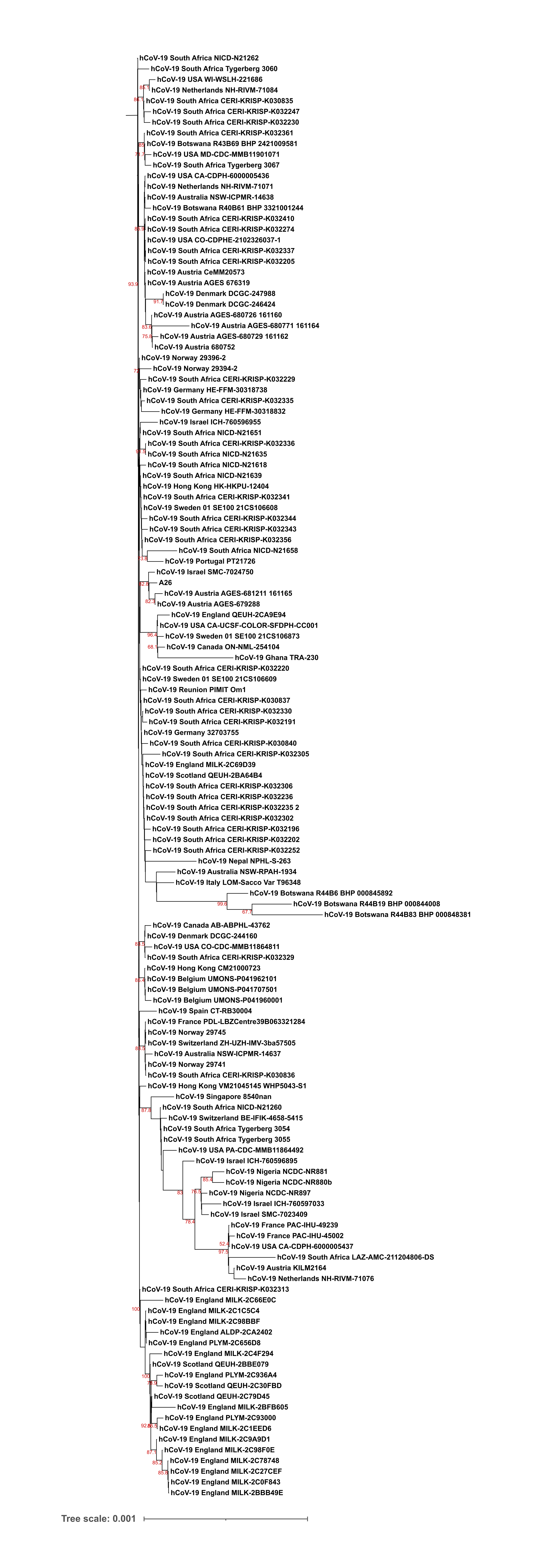

Supplement: Supplementary file 1 [file microorganisms-10-01676-s001.zip › Figure S1.png]
